# Supplementary material for: Diapause Regulation in Newly Invaded Environments: Termination Timing Allows Matching Novel Climatic Constraints in the Box Tree Moth, Cydalima perspectalis (Lepidoptera: Crambidae)
Source: Insects. 2020 Sep 12;11(9):629. doi: 10.3390/insects11090629 (PMC7563306; doi:10.3390/insects11090629)
Supplement: Supplementary file 1 [file insects-11-00629-s001.pdf]

Table S1. After five weeks of exposure to phase B (Temperature:Photoperiod conditions), unopened cocoons were transferred for one month to summer conditions (20 °C, 16:8 L:D). This table summarizes the number of individuals transferred (N) and the percentage of which that resumed their activity (Perc.).

**Temperature (°C)**		**5**				**10**				**15**				**20**			
Larval instar		L3		L4		L3		L4		L3		L4		L3		L4	
		N	Perc.	N	Perc.	N	Perc.	N	Perc.	N	Perc.	N	Perc.	N	Perc.	N	Perc.
Photoperiod (L:D)	8:16	14	57.14	8	75.00	6	0.00	3	33.33	5	0.00	0	0.00	0	0.00	0	0.00
	10:14	14	35.71	14	14.29	8	0.00	6	0.00	6	0.00	10	0.00	2	0.00	2	0.00
	12:12	7	71.43	16	75.00	5	0.00	14	0.00	1	0.00	0	0.00	0	0.00	3	0.00
	14:10	7	14.29	16	6.25	14	0.00	2	50.00	0	0.00	0	0.00	0	0.00	0	0.00
	16:8	18	61.11	27	100.00	7	0.00	9	11.11	8	0.00	8	0.00	0	0.00	0	0.00
